# Immune response in dogs with myxomatous mitral valve disease: insights into monocyte and lymphocyte subtypes and natural killer cells

**DOI:** 10.1093/jvimsj/aalag028

**Published:** 2026-02-25

**Authors:** Martina Cimerman, Alenka Nemec Svete, Anže Baš, Melita Hajdinjak, Miha Bajc, Sara Petek, Katka Pohar, Alojz Ihan, Uroš Krapež, Branko Krt, Klemen Dolinar, Katarina Miš, Sergej Pirkmajer, Aleksandra Domanjko Petrič

**Affiliations:** Small Animal Clinic, Veterinary Faculty, University of Ljubljana, Gerbičeva 60, 1000 Ljubljana, Slovenia; Small Animal Clinic, Veterinary Faculty, University of Ljubljana, Gerbičeva 60, 1000 Ljubljana, Slovenia; Faculty of Education, University of Ljubljana, 1000 Ljubljana, Slovenia; University of Ljubljana, Faculty of Electrical Engineering, Laboratory of Applied Mathematics and Statistics, Tržaška c. 25, 1000 Ljubljana, Slovenia; Institute of Microbiology and Immunology, Faculty of Medicine, University of Ljubljana, Vrazov trg 2, 1000 Ljubljana, Slovenia; Institute of Microbiology and Immunology, Faculty of Medicine, University of Ljubljana, Vrazov trg 2, 1000 Ljubljana, Slovenia; Institute of Microbiology and Immunology, Faculty of Medicine, University of Ljubljana, Vrazov trg 2, 1000 Ljubljana, Slovenia; Institute of Microbiology and Immunology, Faculty of Medicine, University of Ljubljana, Vrazov trg 2, 1000 Ljubljana, Slovenia; Institute of Poultry, Birds, Small Mammals and Reptiles, Veterinary faculty, University of Ljubljana, 1000 Ljubljana, Slovenia; Institute of Microbiology and Parasitology, Veterinary faculty, University of Ljubljana, Gerbičeva 60, 1000 Ljubljana, Slovenia; Institute of Pathophysiology, Faculty of Medicine, University of Ljubljana, Vrazov trg 2, 1000 Ljubljana, Slovenia; Institute of Pathophysiology, Faculty of Medicine, University of Ljubljana, Vrazov trg 2, 1000 Ljubljana, Slovenia; Institute of Pathophysiology, Faculty of Medicine, University of Ljubljana, Vrazov trg 2, 1000 Ljubljana, Slovenia; Small Animal Clinic, Veterinary Faculty, University of Ljubljana, Gerbičeva 60, 1000 Ljubljana, Slovenia

**Keywords:** canine, congestive heart failure, flow cytometry, monocyte subtypes, natural killer cells, t regulatory cells, cytokines

## Abstract

**Background:**

The immune response in dogs with myxomatous mitral valve disease (MMVD) is understudied.

**Hypothesis/Objectives:**

Investigate the populations of monocyte subtypes, natural killer cells (NK cells), and T lymphocyte subtypes in dogs with MMVD and their relationships with selected cytokines and echocardiographic parameters at different stages of the disease and in comparison to healthy dogs.

**Animals:**

Eighty-one client-owned dogs: 64 with MMVD (preclinical stage, compensated congestive heart failure [CHF], decompensated CHF) and 17 healthy dogs.

**Methods:**

Cross-sectional study. Natural killer (NK) cells, monocyte subtypes, T lymphocyte subtypes, and B lymphocytes were identified using flow cytometry. Generalized linear models were used to compare variables between groups. Pairwise comparisons were performed using estimated marginal means with Tukey correction. *P*-value < .05 was considered significant.

**Results:**

The percentage of monocytes was higher in dogs with compensated and decompensated CHF compared with preclinical dogs. The percentage of activated T helper lymphocytes was lower in compensated CHF compared with all other groups. No differences in percentages were found for monocyte subtypes, NK cells, cytotoxic, regulatory and double positive T lymphocytes, or B lymphocytes. Concentration of monocyte chemoattractant protein-1 was higher in the decompensated CHF compared with all other groups, and the concentration of keratinocyte chemotactic-like chemokine was higher in the decompensated CHF compared with the preclinical group.

**Conclusions and clinical importance:**

Higher concentrations and percentages of total monocytes and concentrations of proinflammatory cytokines in CHF suggest an inflammatory pathway in MMVD progression. Activated T helper lymphocytes may be downregulated in dogs with compensated CHF.

## Introduction

Myxomatous mitral valve disease (MMVD) is the most common acquired heart disease in dogs and can progress to congestive heart failure (CHF) and death.^1^ Systemic inflammation, marked by increased neutrophils, monocytes, cytokines, and inflammatory biomarkers, has been associated with the onset and progression of CHF.^2–4^

In dogs with MMVD, disease progression is associated with alterations of T lymphocyte (CD3^+^) subtypes, notably an inverted ratio of T helper lymphocytes (CD3^+^CD4^+^) to cytotoxic T lymphocytes (CD3^+^CD8^+^) and increased percentage of regulatory T lymphocytes (CD4^+^FoxP3^+^CD25^+^).^5,6^ Additionally, higher CD3^+^CD8^+^ lymphocyte concentration and lower CD3^+^CD4^+^ lymphocyte percentage were linked to worse survival outcomes.^7^

Natural killer cells (NK cells) were found to be protective against the development of cardiac fibrosis, and studies in both mice and humans have identified decreased numbers of NK cells in cardiovascular disease.^8,9^

In human medicine, monocyte subtypes have been suggested to contribute to the inflammatory response in CHF, influencing cardiac remodeling and fibrosis via cytokine and chemokine release.^10,11^

The renin-angiotensin-aldosterone axis and sympathetic nervous system have been associated with immune pathway activation, suggesting that the immune system may play a role in CHF progression.^12^

Further research into understanding immune mechanisms may help identify new approaches that potentially could improve survival, management, and quality of life in patients with CHF. We aimed to characterize immune regulation across MMVD stages by analyzing NK cells, monocyte subtypes, and T lymphocyte subtypes.

We hypothesized that dogs with MMVD would have increased percentages of cytotoxic and regulatory T lymphocytes, decreased percentages of NK cells and T helper lymphocytes, and altered monocyte subtypes. These immune changes were expected to correlate with increased concentrations of inflammatory cytokines, C-reactive protein (CRP), and the cardiac biomarker N-terminal natriuretic peptide type B (NT-proBNP) as the disease progresses.

## Materials and methods

### Study population

Of 253 dogs screened, 81 met the inclusion criteria for this cross-sectional study: 64 with MMVD at various stages and 17 healthy dogs. Dogs were presented to the cardiology department of the University Small Animal Clinic between September 2022 and January 2024 for heart murmur evaluation, clinical signs of CHF or routine health screening.

All dog owners signed an informed consent form before participating in the study.

All procedures complied with current government regulations (Animal Protection Act, the Official Gazette, 43/2007). The Animal Welfare Committee approved this study (license no. 25-01/2023-1; January 25, 2023).

Included dogs were classified according to American College of Veterinary Internal Medicine (ACVIM) guidelines^1^ into preclinical (B2), compensated (C), and decompensated CHF (C and D).

Dogs with systemic diseases (eg, endocrine, metabolic, neoplastic, or inflammatory conditions) or those treated with immunosuppressants or antibiotics in the previous six months were excluded.

Healthy small breed dogs were selected during routine health visits. Prerequisites for the inclusion of healthy dogs required normal clinical examinations, echocardiography, and laboratory results, with a minimum age of 5 years to match the patient group.

All dogs underwent clinical examinations, echocardiography, and electrocardiography. Thoracic radiography was performed in dogs with suspected CHF (eg, tachypnea, dyspnea) or coughing. The ACVIM B2 classification required left ventricular end-diastolic diameter in M-mode, normalized for body weight (cm/kg^0.299^); (LVIDdN) > 1.7, left atrial to aortic ratio (LA/Ao) > 1.6 and left atrial diameter in long axis right parasternal view normalized for body weight (cm/kg^0.309^; LAN) > 1.65.^1,13^ Clinically stable or compensated patients had a history of CHF but were asymptomatic at rest with normal heart and respiratory rates, and no radiographic signs of pulmonary edema. Decompensated patients had tachycardia, tachypnea, dyspnea, or some combinations of these at rest, radiographic evidence of cardiogenic pulmonary edema and often required hospitalization. Dogs with refractory CHF (ACVIM D) were grouped with active CHF cases (ACVIM C) as decompensated or clinically unstable because of frequent treatment adjustments or hospitalizations.^14^

Clinical staging and diagnostic testing, including echocardiography (2-Dimensional, M-mode, color, Doppler; GE Vivid E9, General Electric Healthcare) were performed by a trained and experienced veterinarian. Echocardiographic parameters used for statistical analysis included: LAN, LVIDdN, left ventricular end-systolic diameter in M-mode, normalized for body weight (cm/kg^0.387^; LVIDsN),^13^ LA/Ao, early mitral inflow wave E (m/s), late mitral inflow wave A (m/s) and their ratio (E/A), and tricuspid regurgitation-derived pressure gradient TRPG (mmHg).

Treatment followed clinicians’ preferences, generally aligned with ACVIM guidelines.^1^ Preclinical dogs received pimobendan or had no treatment, whereas CHF cases were treated with combinations of pimobendan, angiotensin-converting enzyme inhibitors, spironolactone, diuretics (furosemide or torsemide or both) and amlodipine, or were untreated at inclusion.

### Blood sampling and processing

Blood was collected for a CBC with white blood cell differential count, biochemistry, flow cytometry, and measurements of NT-proBNP, cytokines, chemokines, and CRP concentrations. Routine tests were performed within 1 h. Plasma and serum samples were stored at −80°C until analysis, and flow cytometry samples were kept at room temperature in the dark and analyzed within 24 h.

Plasma NT-proBNP (pmol/L) was measured using IDEXX ELISA (IDEXX BioAnalytics Europe, Leipzig, Germany) and serum CRP concentration (ng/mL) using a VetLine Canine CRP ELISA kit (Gold Standard Diagnostics, Germany), following manufacturers’ protocols.

### Flow cytometry

Multicolor flow cytometry was used to measure the percentages of total monocytes (CD11b^+^) and their subtypes (CD14^+^MHC-II^−^, CD14^+^MHC-II^+^, CD14^−^MHC-II^+^),^15^ NK cell populations characterized in 3 ways (NK1, CD21^−^CD5^−^ CD3^−^; NK2, CD21^−^CD5^dim^ CD3^−^; NK3, CD21^−^CD5^dim^CD3^+^),^16–18^ percentages of total CD3^+^ lymphocytes and their activated form using the CD25 marker of activation (CD3^+^ CD25^+^), and their subtypes (T helper cells (CD3^+^CD4^+^), activated T helper cells (CD3^+^CD4^+^CD25^+^), cytotoxic T cells (CD3^+^CD8^+^), activated cytotoxic T cells (CD3^+^CD8^+^CD25^+^), regulatory T cells (CD4^+^FoxP3^+^CD25^+^), double negative T lymphocytes (DNT, CD4^−^CD8^−^), double positive T lymphocytes (DPT, CD4^+^CD8^+^), and percentages of B lymphocytes (CD3^−^CD21^+^) lymphocytes.^5,6^

The gating strategy for the investigated cells is shown in Figures S1-S4 for each individual dog group: healthy (Figure S1), preclinical group (Figure S2), compensated CHF (Figure S3), decompensated CHF (Figure S4).

Monoclonal anti-mouse and anti-rat antibodies were used for labeling against CD3 (clone CA17.2A12, FITC, Cat. No. MA516605), CD4 (clone YKIX302.9, PE-Cyanine7, Cat. No. 25-5040-42), CD5 (clone YKIX322.3, PE, Cat. No. 12-5050-42), CD8 (clone YCATE55.9, APC, Cat. No. 17-5080-42), CD11b (clone M1/70, PE, Cat. No. 12-0112-82), CD14 (clone TüK4, FITC, Cat. No. MA182074), CD21 (clone LT21, Alexa Fluor 647, Cat. No. MA518131), CD25 (clone P4A10, PE, Cat. No. 12-0250-42), CD45 (clone YKIX716.13, eFluor 450, Cat No. 48-5450-42), MHC-II (Clone YKIX334.2, APC, Cat. No. 17-5909-42), and FoxP3 (clone FJK-16 s, APC, Cat. No. 17-5773-82). All monoclonal antibodies were manufactured by eBioscience, San Diego, CA, USA.

#### Extracellular staining

Four tubes with 100 μL of whole blood were prepared and stained with monoclonal antibodies for cell surface markers, then incubated for 30 min at 2-8°C in the dark. The first tube was stained for CD3^+^ lymphocytes and their subtypes with 5 μL CD45 eFluor 450, 5 μL CD4 PE-Cyanine7, 5 μL CD25 PE, 5 μL CD8 APC, and 10 μL CD3 fluorescein isothiocyanate (FITC). T regulatory lymphocytes were stained in a separate tube with 5 μL CD45 eFluor 450, 5 μL CD4 PE-Cyanine7, 5 μL CD25 PE, and 10 μL CD3 FITC. The third tube for B lymphocytes and NK cells was stained with 5 μL CD45 eFluor 450, 10 μL CD3 FITC, 5 μL CD5 PE, and 4 μL CD21 Alexa Fluor 647. The last tube was stained for monocytes and their subtypes with 5 μL CD45 eFluor 450, 5 μL MHC II APC, 10 μL CD14 FITC, and 0.63 μL CD11b PE. The erythrocytes were lysed with 1% formaldehyde (FACS Lysing solution; Cat. No. 349202) and incubated for 10 min at room temperature in the dark. The samples were centrifuged at 480 × *g* for 5 min at room temperature, and the supernatant was discarded. Tubes were washed with 1% bovine serum albumin (BSA; Sigma Aldrich, Cat. No. A9418) in phosphate-buffered saline (PBS; sodium chloride: Supelco, Cat. No. 1.06404.1000; disodium hydrogen phosphate dihydrate, Honeywell—Fluka, Cat. No. 30435; potassium dihydrogen phosphate, Sigma Aldrich, Cat. No. 60220; 1% BSA in PBS), resuspended in 1% BSA in PBS and analyzed using the flow cytometer.

#### Intracellular staining

For intracellular staining of CD4^+^FoxP3^+^CD25^+^ lymphocytes, the procedure was continued after extracellular staining using the FoxP3/Transcription Factor Staining Buffer Set (eBioscience, Cat. No. 00-5523-00) according to the manufacturer’s recommended protocol. Cells were fixed with fixation buffer and incubated in the dark at 2-8°C for 30 min and washed twice with 1× permeabilization buffer. FoxP3 was added, and the samples were incubated for 30 min in the dark at room temperature. After incubation, the cells were washed twice with 1× permeabilization buffer, resuspended in 1% BSA in PBS, and analyzed using flow cytometry.

All samples were analyzed using a FACSCanto II flow cytometer (BD Biosciences, San Jose, California) using FACSDiva software, version 8.0.1 (BD Biosciences).

### Cytokines and chemokines

Serum cytokines and chemokines (Interleukin-2 (IL-2), Interleukin-6 (IL-6), Interleukin-10 (IL-10), Tumor necrosis factor α (TNF-α), Keratinocyte-derived chemokine-like (KC-like), Monocyte chemoattractant protein-1 (MCP-1)) were measured using a MILLIPLEX magnetic bead-based multiplex immunoassay (MAP canine Cytokine/Chemokine Panel, Millipore, Darmstadt, Germany) on a Luminex MAGPIX with xPONENT 4.2 software for interpretation. Assays followed manufacturers’ protocols, including 7-point standard curves, quality control samples, and duplicate measurements.

Concentration of IL-1β was measured using a canine-specific ELISA kit (Millipore). The absorbance was read at 450 nm using a Sunrise microplate reader (Tecan, Männedorf, Switzerland) and the results were interpreted using Magellan and CurveExpert 1.4 software.

### Statistical analysis

Data were analyzed using R version 4.3.3^19^ and Jamovi project.^20^ The normality of each of the continuous variables was assessed using the Shapiro–Wilk test in combination with Q-Q plots, with parametric tests or nonparametric tests applied accordingly. Considering the predominantly non-normally distributed data and aiming for clarity, all graphs illustrate the median and interquartile range (IQR). Summary statistics for left-censored data (cytokines) were computed using robust regression on order statistics with the censorplot function in the contributed NADA package in R^21^ cenboxplot function, and as a result the boxplots for cytokines incorporate regression on order statistics (ROS)-estimated values below the detection limit. Detection limits (DL) are indicated within the graphs. For the purposes of inferential statistical analysis observations outside the detection limit were assigned the value of the lower limit and nonparametric tests were performed, as proposed previously.^22^ Initially, one-way analysis of variance (ANOVA) or Kruskal-Wallis tests were applied to compare groups for each parameter. Given the number of comparisons and to mitigate the false discovery rate, the Benjamini–Hochberg method was employed to adjust *P*-values. Subsequently the adjusted *P*-values were used as the basis for determining the necessity of post-hoc testing. Accordingly, the Kruskal–Wallis test and Fisher’s exact test were used to assess differences in the distribution of potential confounding factors, such as sex ratio, weight, and age, between the groups. Because possible confounding effects were present, we applied a generalized linear or censored regression model to account for sex and age differences. Pairwise comparisons were conducted using estimated marginal means. Wald test statistics (t or z, depending on the model) and *P*-values were obtained from the fitted models, with *P*-values adjusted using Tukey’s method for multiple comparisons. For outcomes modeled on the original scale and meeting normality assumptions, group differences are presented as absolute differences in means with 95% CIs. For outcomes with skewed distributions, appropriate transformations were applied to satisfy model assumptions. To aid interpretation, estimated group differences were back-transformed to the original measurement scale and are reported as geometric mean ratios (GMRs), reflecting relative differences rather than absolute ones. A more detailed description of the models, including parameter estimates, is provided in the Supplementary Material 1. The absolute magnitude of the observed correlation ρ was interpreted as follows: 0.00-0.10: negligible correlation; 0.10-0.39: weak correlation; 0.40-0.69: moderate correlation; 0.70-0.89: strong correlation; 0.90-1.00: very strong correlation.^23^ Significance was set at *P* < .05.

## Results

The study included 81 dogs: 64 dogs with MMVD (23 preclinical, 22 dogs compensated CHF, 19 decompensated CHF) and 17 healthy dogs. Demographic data are summarized in Table 1.

**Table 1:** Demographic data of dogs included in the study.

**Group**	**Preclinical**	**Stable CHF**	**Unstable CHF**	**Control**
**Number**	23	22	19	17
**Sex** ^a^ **(female/male)**	9/14	6/16	7/12	13/4
**Age (years)** ^a^				
**Median**	10.6	11.4	12.2	6.8
**IQR**	8.2-12.8	10.3-13.1	9.2-12.7	5. 6-7.8
**Weight (kg)**				
**Median**	10.7	7.3	7.5	6.8
**IQR** **Breeds**	7.0-12.4 1 BIG, 1 BRS, 10 CKCS, 1 JRT, 1 MPO, 1 MS, 5 M, 1 PA, 1 TS, 1 WH	5.6-9.5 3 χ, 3 CKCS, 1 DH, 3 M, 1 ML, 1 JRT, 1 MP, 3 MPO, 2 SHI, 2 TS, 1 VIZ, 1 WH	6.2-11.6 1 BIG, 4 χ, 6 CKCS, 1 GSP, 1 JCH, 3 M, 1 ML, 1 PEK, 1 SHI	5.5-7.7 1 BAS, 1 CKCS, 2 JRT, 3 M, 2 ML, 1 MP, 1 MPO, 1 RWD, 1 SHE, 1 SHI, 3 TS

^a^Indicates a statistically significant difference between the MMVD group and the healthy control group (*P* < .05).

Abbreviations: BAS = Basenji; BIG = Beagle; BRS = Brittany spaniel; CHF = congestive heart failure; χ = Chihuahua; CKCS = Cavalier King Charles Spaniel; DH = Dachshund; GSP = German Shorthaired Pointer; IQR = interquartile range (25th-75th percentile); JCH = Japanese Chin; JRT = Jack Russell Terrier; M = mixed breed; ML = Maltese; MP = Miniature Pinscher; MPO = Miniature Poodle; MS = Miniature Schnauzer; PA = Papillon; PEK = Pekingese; RWD = Romanian water dog; SHE = Shetland Sheepdog*;* SHI = Shih-Tzu; TS = Tibetan spaniel; VIZ = Vizsla; WH = Whippet.

No weight differences were found between groups of patients (*P* = .08), but sex ratios (*P* = .02, Fisher’s exact test) and age (*P* < .001) differed significantly, with the healthy group being skewed female and significantly younger (*P* < .001) than MMVD groups.

**Figure 1:**
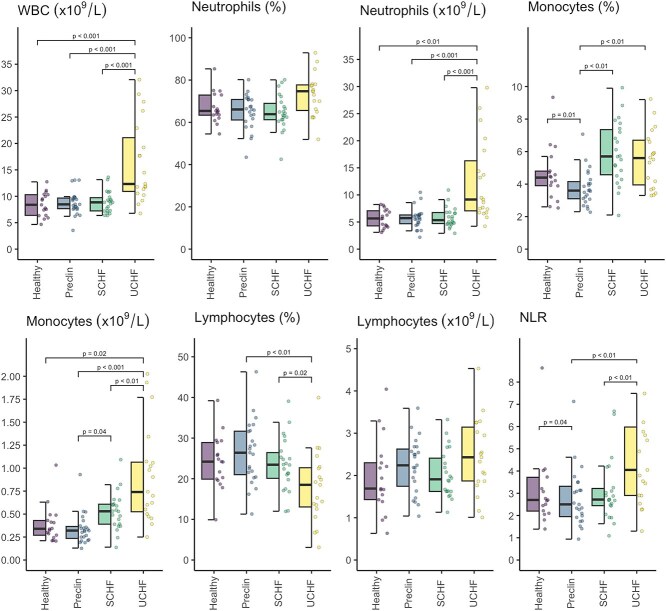
Concentrations and percentages of selected blood cells measured with hematologic analyzer in various stages of myxomatous mitral valve disease and healthy dogs. Abbreviations: NLR = neutrophil to lymphocyte ratio; Preclin = preclinical group; SCHF = compensated congestive heart failure group; UCHF = decompensated congestive heart failure group; WBC = white blood cell count.

Concentrations and percentages of selected blood cells measured using a hematological analyzer are presented in Figure 1. Dogs in decompensated CHF had 68% higher white blood cell count (WBC) than the healthy group (GMR = 1.68; 95% CI, 1.21-2.33; Wald *t* = 4.19; degrees of freedom [df] = 75; *P* < .001), 75% higher than the preclinical group (GMR = 1.75; 95% CI, 1.35-2.26; Wald *t* = 5.70; df = 75; *P* < .001), and 73% higher than the compensated CHF group (GMR = 1.73; 95% CI, 1.33-2.25; Wald *t* = 5.51; df = 75; *P* < .001). Neutrophil concentrations in the decompensated CHF group were approximately twice those of both the preclinical group (GMR = 1.96; 95% CI, 1.43-2.69; Wald *t* = 5.58; df = 75; *P* < .001) and compensated CHF group (GMR = 1.98; 95% CI, 1.44-2.74; Wald *t* = 5.60; df = 75; *P* < .001) and were 1.7-fold higher in comparison with the healthy group (GMR = 1.71; 95% CI, 1.15-2.56; Wald *t* = 3.53; df = 75; *P* = .004). The percentage of monocytes was 44% higher in the compensated group (GMR = 1.44; 95% CI, 1.13-1.82; Wald *t* = 4.01; df = 75; *P* = .001) and 41% higher in the decompensated CHF group (GMR = 1.41; 95% CI, 1.11-1.79; Wald *t* = 3.72; df = 75; *P* = .002) compared with the preclinical group, whereas the monocyte percentage was 29% lower in the preclinical group than in the healthy group (GMR = 0.71; 95% CI, 0.53-0.95; Wald *t* = −3.13; df = 75; *P* = .01). In addition, the monocyte concentration in dogs with decompensated CHF was 69% higher than in the healthy group (GMR = 1.69; 95% CI, 1.07-2.67; Wald *t* = 3.00; df = 75; *P* = .02), 70% higher than in the compensated CHF group (GMR = 1.70; 95% CI, 1.18-2.46; Wald *t* = 3.79; df = 75; *P* = .002) and 2.4 times higher than in the preclinical group (GMR = 2.45; 95% CI, 1.70-3.51; Wald *t* = 6.49; df = 75; *P* < .001). Monocyte concentration also was 44% higher in the compensated CHF group than in the preclinical group (GMR = 1.44; 95% CI, 1.01-2.04; Wald *t* = 2.69; df = 75; *P* = .04). The percentage of lymphocytes was, on average, 7.8% higher in the preclinical group (95% CI: 2.0-13.6%; Wald *t* = 3.53; df = 75; *P* = .004) and 6.6% higher in the compensated CHF group (95% CI, 0.7-12.5%; Wald *t* = 2.94; df = 75; *P* = .02) than in the decompensated CHF group. The neutrophil to lymphocyte ratio (NLR) was 74% higher in the decompensated CHF group than in the preclinical group (GMR = 1.74; 95% CI, 1.16-2.63; Wald *t* = 3.57; df = 75; *P* = .003) and 76% higher than in the compensated CHF group (GMR = 1.76; 95% CI, 1.16-2.67; Wald *t* = 3.58; df = 75; *P* = .003).

**Figure 2:**
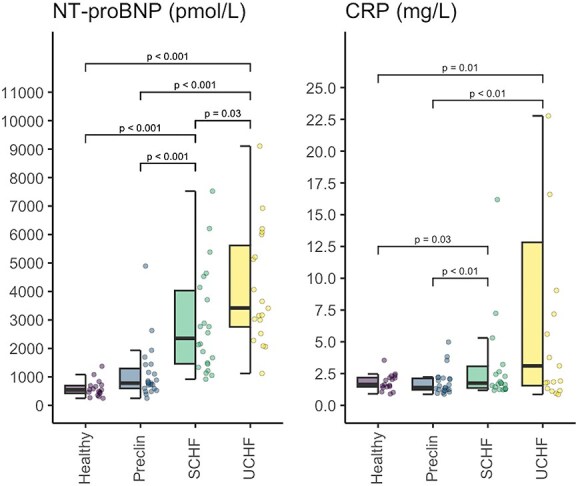
N-terminal natriuretic peptide type B and C-reactive protein concentrations. Abbreviations: CRP = C-reactive protein; NT-proBNP = N-terminal natriuretic peptide type B; Preclin = preclinical group; SCHF = compensated congestive heart failure group; UCHF = decompensated congestive heart failure group.

The concentrations of NT-proBNP and CRP are presented in Figure 2. The concentration of NT-proBNP was almost four-fold higher in the decompensated CHF group than in the preclinical group (GMR = 3.92; 95% CI, 2.51-6.13; Wald *t* = 8.06; df = 75; *P* < .001), 63% higher than in the compensated CHF group (GMR = 1.63; 95% CI, 1.03-2.56; Wald *t* = 2.82; df = 75; *P* = .03) and approximately 4.8 times higher than in the healthy group (GMR = 4.78; 95% CI, 2.73-8.40; Wald *t* = 7.31; df = 75; *P* < .001). Additionally, NT-proBNP concentration in the compensated CHF group was more than twice as high as in the preclinical group (GMR = 2.41; 95% CI, 1.56-3.73; Wald *t* = 5.28; df = 75; *P* < .001) and nearly three-fold higher than in the healthy group (GMR = 2.95; 95% CI, 1.64-5.29; Wald *t* = 4.86; df = 75; *P* < .001).

The concentration of CRP was more than eight-fold higher in the decompensated CHF group compared with the preclinical group (GMR = 8.47; 95% CI, 1.93-37.30; Wald *z* = 3.71; *P* = .002) and approximately 7.9 times higher than in the healthy group (GMR = 7.87; 95% CI, 1.60-38.67; Wald *z* = 3.33; *P* = .01). In the compensated CHF group, CRP concentrations were 4.3 times higher than those in the preclinical group (GMR = 4.27; 95% CI, 1.52-12.00; Wald *z* = 3.61; *P* = .002), and approximately four-fold higher than in the healthy group (GMR = 3.98; 95% CI, 1.17-13.56; Wald *z* = 2.90; *P* = .03).

**Figure 3:**
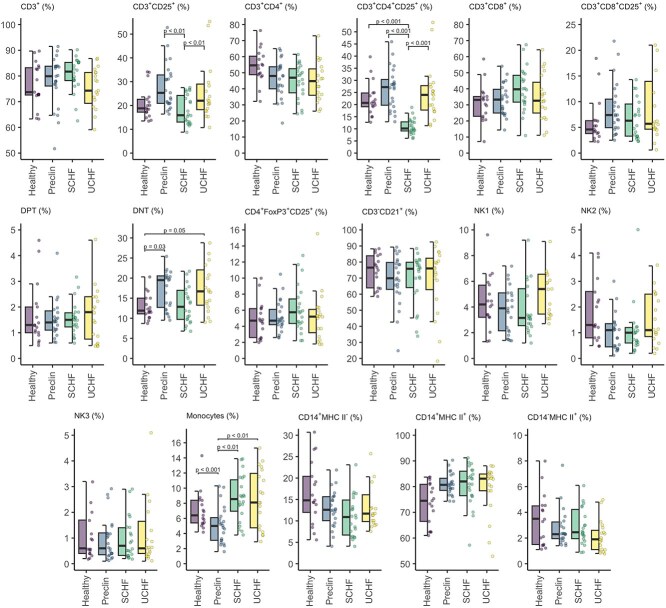
Selected immune cells measured by flow cytometry in various stages of myxomatous mitral valve disease and healthy dogs. Abbreviations: CD3^+^ = T lymphocytes; CD3^+^CD25^+^ = activated T lymphocytes; CD3^+^CD4^+^ = T helper lymphocytes; CD3^+^CD4^+^CD25^+^ = activated T helper lymphocytes; CD4^+^FoxP3^+^CD25^+^ = regulatory T lymphocytes; CD3^+^CD8^+^ = cytotoxic T lymphocytes; CD3^+^CD8^+^CD25^+^ = activated cytotoxic T lymphocytes; CD3^−^CD21^+^ = B lymphocytes; CD14^+^MHC II^−^ = monocyte subtype 1; CD14^+^MHC II^+^ = monocyte subtype 2; CD14^−^MHC II^+^ = monocyte subtype 3; DNT = double negative T lymphocytes; DPT = double positive T lymphocytes; NK1 = natural killer cell population 1 (CD21^−^CD5^−^CD3^−^); NK2 = natural killer cell population 2 (CD21^−^CD5^dim^CD3^−^); NK3 = natural killer cell population 3 (CD21^−^CD5^dim^CD3^+^); Preclin = preclinical group; SCHF = compensated congestive heart failure group; UCHF = decompensated congestive heart failure group.

Proportions of selected immune cells in MMVD groups and healthy dogs measured by flow cytometry are presented in Figure 3. The percentage of CD3^+^CD25^+^ lymphocytes was 38% lower in the compensated CHF group than in the preclinical group (GMR = 0.62; 95% CI, 0.47-0.82; Wald *t* = −4.43; df = 75; *P* = .001), whereas it was 44% higher in the decompensated CHF group compared with the compensated CHF group (GMR = 1.44; 95% CI, 1.08-1.93; Wald *t* = 3.28; df = 75; *P* = .01).

The percentage of CD3^+^CD4^+^CD25^+^ lymphocytes in the compensated CHF group was less than half of that in the healthy (GMR = 0.41; 95% CI, 0.29-0.59; Wald *t* = −6.60; df = 75; *P* < .001) and in the preclinical group (GMR = 0.40; 95% CI, 0.31-0.52; Wald *t* = −9.14; df = 75; *P* < .001) and the decompensated CHF group (GMR = 0.45; 95% CI, 0.34-0.59; Wald *t* = −7.68; df = 75; *P* < .001). No significant difference was found in the percentages of DPT, whereas the percentage of DNT was 45% higher in preclinical dogs compared with healthy dogs (GMR = 1.45; 95% CI, 1.02-2.07; Wald *t* = 2.80; df = 75; *P* = .03).

The percentage of total monocytes (CD11b^+^) was 60% higher in both the compensated (GMR = 1.58; 95% CI, 1.17-2.14; Wald *t* = 3.94; df = 75; *P* = .001) and decompensated CHF group (GMR = 1.59; 95% CI, 1.17-2.18; Wald *t* = 3.94; df = 75; *P* = .001) in comparison with the preclinical group. Additionally, the preclinical group had a 50% lower percentage of total monocytes than the healthy group (GMR = 0.50; 95% CI, 0.35-0.73; Wald *t* = −4.88; df = 75; *P* < .001).

No significant differences were found in the percentages of different monocyte subtypes, natural killer (NK) cells, cytotoxic and regulatory T lymphocytes, or B lymphocytes between the groups.

**Figure 4:**
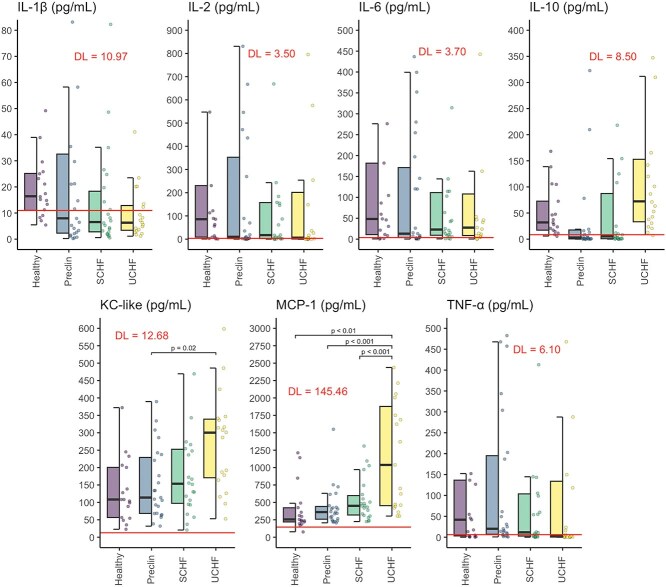
Selected cytokine and chemokine concentrations in various stages of myxomatous mitral valve disease and healthy dogs. Abbreviations: IL = interleukin; KC-like = keratinocyte chemotactic-like; MCP-1 = monocyte chemoattractant protein 1; Preclin = preclinical group; SCHF = compensated congestive heart failure group; TNF-α = tumor necrosis factor α; UCHF = decompensated congestive heart failure group; red line denotes the limit of detection.

The concentrations of cytokines and chemokines are shown in Figure 4. The concentrations of MCP-1 and KC-like were quantifiable in all dogs, whereas concentrations of IL-1β could not be quantified in 54% of cases, IL-2 in 39%, IL-6 in 21%, IL-10 in 68%, and TNF-α in 51% of cases because they were below the DL. The highest DL is drawn as the red horizontal line in Figure 4 and all entries below that line are ROS-estimated values.

The concentration of KC-like was, on average, 114.6 pg/mL higher in dogs with decompensated CHF than in preclinical dogs (95% CI, 18.3-210.9 pg/mL; Wald *z* = 3.06; *P* = .02). The concentration of MCP-1 was, on average, 848.0 pg/mL higher in the decompensated CHF group than in the preclinical group (95% CI: 398.8-1297.8 pg/mL; Wald *z* = 4.85; *P* < .001), 791.9 pg/mL higher than in the compensated CHF group (95% CI: 335.6-1248.3 pg/mL; Wald *z* = 4.46; *P* < .001) and 778.6 pg/mL higher than in the healthy group (95% CI, 206.9-1347.3 pg/mL; Wald *z* = 3.52; *P* = .004). No significant differences were found in other cytokines investigated between the groups.

In addition, we examined the correlations between selected immune cells and cytokines as well as selected echocardiographic parameters across all stages of MMVD.

We found a negative correlation between CD14^−^MHC-II^+^ monocyte subtype and MCP-1 (ρ = −0.41, *P* < .001), whereas a positive correlation was found between total monocytes (CD11b^+^) and MCP-1 (ρ = 0.27, *P* = .03). Also a negative correlation was found between CD3^+^CD4^+^ lymphocytes and IL-6 (ρ = −0.28, *P* = .03) and TNF-α (ρ = −0.28, *P* = .024), whereas CD3^+^CD8^+^ lymphocytes were positively correlated with TNF-α (ρ = 0.27, *P* = .03; Figure S5).

N-terminal natriuretic peptide type B concentrations were positively correlated with both total monocyte percentage (ρ = 0.35, *P* = .01) and MCP-1 (ρ = 0.37, *P* = .003). Additionally, CRP concentration showed a positive correlation with total monocyte percentage (ρ = 0.36, *P* = .003) and MCP-1 concentrations (ρ = 0.37, *P* = .003). On the other hand, NT-proBNP was negatively correlated with CD3^+^CD4^+^CD25^+^ lymphocytes (ρ = −0.28, *P* = .02; Figure S6).

The CD14^−^MHC-II^+^ monocyte subtype was positively correlated with LVIDsN (ρ = 0.31, *P* = .011), whereas the CD14^+^MHC-II^+^ monocyte subtype was positively correlated with LA/Ao (ρ = 0.26, *P* = .04). The total percentage of monocytes (CD11b^+^) was positively correlated with LAN (ρ = 0.25, *P* = .05). The NK3 characterization of NK cells was positively correlated with E/A (ρ = 0.27, *P* = .03), whereas the NK2 subtype was positively correlated with tricuspid regurgitation-derived pressure gradient (TRPG) (ρ = 0.28, *P* = .04). B lymphocytes were positively correlated with E/A (ρ = 0.25, *P* < .05). The percentage of CD4^+^FoxP3^+^CD25^+^ lymphocytes was negatively correlated with LVIDsN (ρ = −0.27, *P* = .03) and E/A (ρ = −0.25, *P* = .05). The percentage of CD3^+^CD8^+^CD25^+^ lymphocytes was positively correlated with E/A (ρ = 0.26, *P* = .04), whereas CD3^+^CD4^+^CD25^+^ lymphocytes were negatively correlated with LA/Ao (ρ = −0.30, *P* = .02). The percentage of CD3^+^CD25^+^ lymphocytes also was negatively correlated with LA/Ao (ρ = −0.30, *P* = .02) and A (ρ = −0.28, *P* = .03; Figure S7).

## Discussion

Our study focused on the role of monocytes and their subtypes, NK cells and CD4^+^FoxP3^+^CD25^+^ cells, in MMVD progression and their associations with cytokines and chemokines.

Monocytes are a diverse group of immune cells involved in host defense, inflammation and tissue repair, and have been implicated in cardiovascular disease pathophysiology and prognosis.^10,24,25^ Increased monocyte numbers have been reported in humans^26^ and dogs^3,4^ with CHF. We found higher total monocyte percentages in compensated and decompensated CHF dogs compared with preclinical dogs. Interestingly, monocyte percentages were lower in preclinical dogs than in healthy dogs. This difference is unlikely a consequence of age, because regression analyses controlled for age. The current scientific understanding of decreased circulatory monocyte percentage is that it may be a result of monocyte migration into tissues, immune exhaustion and dysregulation, or the influence of medical treatment.^27,28^ This mobilization out of circulation can lower circulating monocyte percentages even if total numbers remain similar or increase slightly.

It is possible that monocytes transform into tissue macrophages^27^ but only studies on MMVD myocardium could prove this hypothesis.

In our study, CD11b^+^ monocytes showed positive correlations with CRP, NT-proBNP, MCP-1, and LAN, which may reflect increasing inflammation and monocyte involvement in the progression of MMVD.

Monocyte chemoattractant protein-1 plays a central role in monocyte mobilization and recruitment to inflammatory sites.^29^ CRP further amplifies this process by inducing MCP-1 expression, thereby promoting monocyte infiltration into the myocardium and potentially contributing to CHF progression.^30^

In humans, monocyte subtypes play distinct roles in cardiovascular disease: classical monocytes (80%-90%) are phagocytic and respond early to inflammation; intermediate monocytes (5%-10%) produce pro-inflammatory cytokines and present antigens; and non-classical monocytes (2%-8%) aid in tissue repair and inflammation resolution.^11,24,31^ Consistent with previous studies in healthy dogs, we found CD14^+^MHC-II^+^ monocytes to be the predominant subtype.^15,32^ Contrary to our expectations, monocyte subtype distributions did not differ between groups. However, CD14^−^MHC-II^+^ percentages correlated positively with LVIDsN, and negatively with MCP-1, suggesting a potential decrease of this subtype with increasing inflammation and disease progression, which is evident in Figure 3, but it did not reach statistical significance.

Monocyte subtype proportions vary in humans with cardiovascular disease and heart failure.^24,25,33^ One study found decreased concentration of classical monocytes but unchanged concentration of intermediate monocytes in chronic heart failure,^34^ whereas another study linked higher concentrations of intermediate monocytes to increased all-cause mortality.^25^ At this time, we cannot align monocyte subtype labeling in dogs with subtypes in humans and additional studies are needed to describe monocyte phenotypic variations and their roles in diseases.^32^

Our results confirmed increased WBC in CHF patients, especially in decompensated CHF, driven by neutrophilia, consistent with previous findings that inflammation is most prominent in advanced CHF.^2,3,14^ Also, in our study, NLR was higher in decompensated CHF compared with preclinical and compensated CHF, aligning with a previous report of increased NLR in advanced MMVD.^35^

Decreased peripheral NK cells have been reported in humans with myocarditis and ischemic heart disease.^9,36^ In our study, NK cell percentages did not differ between groups. Nevertheless, NK3 cells were positively correlated with E/A, and NK2 cells with TRPG across MMVD stages, which might implicate them in disease progression. NK cells may play both protective and harmful roles in cardiovascular disease by influencing inflammation, tissue injury, and cardiac remodeling.^9^ Canine NK cells are not yet well characterized, with no species-specific marker identified to date. During characterization, based on their expression of surface markers CD3 and CD5, we noticed 2 distinct CD3^+^CD5^+^ populations, one of which was CD4-positive and the other CD8-positive. Given this observation, we hypothesized that the CD3^+^CD5^+^ population may represent natural killer T cells, a CD3^+^ subpopulation with similar functional properties to CD3^+^ and NK cells.^37^

Although NK cells did not differ between MMVD and healthy dogs, their correlation with cardiac inflammation in humans^9^ and disease progression markers in our study emphasizes the need for further investigation into their role in MMVD.

We additionally examined CD3^+^ lymphocyte subtypes, focusing on CD4^+^FoxP3^+^CD25^+^ cells, the percentages of which did not differ between groups but showed negative correlations with LVIDsN and E/A, suggesting their possible role in disease progression.

In humans with CHF, CD4^+^FoxP3^+^CD25^+^ lymphocytes were decreased,^38,39^ and inversely associated with left ventricular end-diastolic diameter and NT-proBNP concentration.^38^

The primary function of CD4^+^FoxP3^+^CD25^+^ lymphocytes is the suppression of excessive immune responses, via anti-inflammatory cytokines, such as IL-10, IL-35 and transforming growth factor β.^40,41^ The dysfunction of CD4^+^FoxP3^+^CD25^+^ may contribute to immune activation in CHF.^38^ Interestingly, unlike humans, one study found increased proportions of these cells across all MMVD stages in dogs.^6^

Studies on CD3^+^CD4^+^ lymphocytes in MMVD are conflicting, one reported lower concentrations,^5^ and another higher concentrations in CHF dogs.^6^ In our study, CD3^+^CD4^+^ lymphocytes did not differ among groups, but the percentage of CD3^+^CD4^+^CD25^+^ lymphocytes was decreased in the compensated CHF group compared with all other groups. This finding differs from another study, that found no differences in CD3^+^CD4^+^CD25^+^ lymphocytes among healthy, preclinical, and CHF groups.^42^ Additionally, in our study CD3^+^CD4^+^ lymphocytes were negatively correlated with IL-6 and TNF-α, and CD3^+^CD4^+^CD25^+^ lymphocytes negatively correlated with NT-proBNP and LA/Ao, suggesting T helper lymphocytes could be downregulated with MMVD progression.

Variable results also have been reported for CD3^+^CD8^+^ lymphocytes in dogs with MMVD; one study reported an increase in CD3^+^CD8^+^ lymphocytes in CHF,^5^ and another a decrease.^6^ We found no differences in the percentage of CD3^+^CD8^+^ and CD3^+^CD8^+^CD25^+^ lymphocytes between the groups. However, CD3^+^CD8^+^ lymphocytes were positively correlated with TNF-α, and CD3^+^CD8^+^CD25^+^ with E/A, suggesting their possible roles with increasing inflammation. Our findings are supported by the results of another study in which CD3^+^CD8^+^ cell concentration was negatively associated with survival and positively correlated with echocardiographic parameters of disease progression, whereas the percentage of CD3^+^CD4^+^ cells was positively associated with survival and negatively correlated with echocardiographic parameters of disease progression in dogs with MMVD.^7^

We also found a higher proportion of DNT lymphocytes in the preclinical group compared with the healthy group, whereas proportions of DPT lymphocytes remained unchanged. One prior study in MMVD dogs showed no group differences in DPT and DNT,^5^ whereas a negative association of these cells with survival was found in dogs with MMVD.^7^ Double negative T lymphocytes are a small CD3^+^ population lacking CD4 and CD8 markers but expressing the T cell receptor.^43^ In humans, they are involved in various chronic inflammatory and autoimmune conditions,^44,45^ with both immunoregulatory (via IL-10) and pro-inflammatory roles that lead to further tissue damage.^45^ Given their ability to modulate immune responses, DNT cells may influence the chronic inflammation observed in CHF and other cardiovascular conditions.

The cytokine and chemokine concentrations were largely unchanged across our groups, apart from MCP-1 and KC-like. The concentration of KC-like was higher in decompensated CHF compared with preclinical dogs, whereas MCP-1 was higher in decompensated CHF compared with all other groups. The lack of differences in other cytokines likely reflects low DLs, similar to a previous study using the same method. In that study, increased MCP-1 but not KC-like concentrations were found in dogs with CHF.^46^ Another study found higher concentrations of KC-like in dogs with CHF compared with healthy dogs and those in preclinical stage of MMVD, but no differences in MCP-1.^47^ They also found a positive correlation between KC-like and cTnI, CRP, haptoglobin, ferritin, WBC, and neutrophils and LA/Ao.^47^ In our study, KC-like was positively correlated with TRPG, and MCP-1 with NT-proBNP, CRP, and monocyte percentage, suggesting a possible role of MCP-1 in MMVD-related inflammatory response and CHF progression.

Our study had some limitations including small numbers of dogs in individual groups. A post-hoc power analysis indicated sufficient power to detect moderate to large effects (power > 0.80), but smaller effects (f^2^ < 0.12) may have been missed. Despite efforts to include more dogs, strict inclusion criteria led to the exclusion of many patients with coexisting inflammatory conditions or asymptomatic MMVD in the healthy group. Additionally, although statistical adjustments were made for age differences between groups, the lack of age-matching still may have influenced immune profiles. Lastly, no technical replicates were performed for flow cytometry analyses, which may have introduced some variability in the findings.

In summary, the increased overall proportion and concentration of monocytes in dogs with CHF and their correlation with markers of disease progression and inflammation could indicate their role in MMVD and CHF pathology. Our results also showed a lower percentage of CD3^+^CD4^+^CD25^+^ cells in compensated CHF compared with all other groups, suggesting downregulation of these cells in compensated CHF. Increased concentrations of MCP-1 and KC-like in decompensated CHF suggest a possible role of these chemokines in the progression of MMVD.

Contrary to our expectations, no differences were found among the groups for monocyte subtypes, NK cells, and cytotoxic and regulatory T lymphocytes. Given the variable results in the human and veterinary medical literature regarding NK and Treg cells, further investigation is warranted.

## Supplementary Material

aalag028_Supplemental_Files

## Data Availability

https://repozitorij.uni-lj.si/IzpisGradiva.php?id=175628.

## Off-label antimicrobial declaration

Authors declare no off-label use of antimicrobials.

## Institutional animal care and use committee or other approval declaration

All procedures complied with the applicable Slovenian governmental regulations (Animal Protection Act, The Official Gazette of the Republic of Slovenia, 43/2007). The Committee for Animal Welfare approved this study, license No.25- 01/2023-1; 25. January, 2023).

## Abbreviations

A: late mitral inflow wave
ACVIM: American College of Veterinary Internal Medicine
1% BSA in PBS: 1% bovine serum albumin in phosphate-buffered saline
CD3^+^: T lymphocytes
CD3^+^CD25^+^: activated T lymphocytes
CD3^+^CD4^+^: T helper lymphocytes
CD3^+^CD4^+^CD25^+^: activated T helper lymphocytes
CD3^+^CD8^+^: cytotoxic T lymphocytes
CD3^+^CD8^+^CD25^+^: activated cytotoxic T lymphocytes
CD4^+^FoxP3^+^CD25^+^: regulatory T lymphocytes
CD3^−^CD21^+^: B lymphocytes
CHF: congestive heart failure
CM: classical monocytes
CRP: C-reactive protein
DNT: double negative T lymphocytes
DPT: double positive T lymphocytes
E: early mitral inflow wave
E/A: early to late mitral flow ratio
IL: interleukin
IM: intermediate monocytes
IQR: interquartile range
KC-like: keratinocyte chemotactic-like
LA/Ao: left atrial to aortic ratio
LAN: left atrial diameter in long axis right parasternal view normalized for body weight (cm/kg^0.309^)
LVIDdN: left ventricular end-diastolic diameter in M-mode, normalized for body weight (cm/kg^0.299^)
LVIDsN: left ventricular end-systolic diameter in M-mode, normalized for body weight (cm/kg^0.387^)
MCP-1: monocyte chemoattractant protein 1
MMVD: myxomatous mitral valve disease
NCM: non-classical monocytes
NK cell: natural killer cell
NLR: neutrophil to lymphocyte ratio
NT-proBNP: N-terminal pro-B-type natriuretic peptide
TNF-α: tumor necrosis factor α
TRPG: tricuspid regurgitation derived pressure gradient
WBC: white blood cell count
