# Supplementary material for: Immune response in dogs with myxomatous mitral valve disease: insights into monocyte and lymphocyte subtypes and natural killer cells
Source: J Vet Intern Med. 2026 Feb 25;40(1):aalag028. doi: 10.1093/jvimsj/aalag028 (PMC12935012; doi:10.1093/jvimsj/aalag028)
Supplement: aalag028_Supplemental_Files [file aalag028_supplemental_files.zip › Supplementary_figure_caption__aalag028.docx]

**Supplementary figure 1:** Representative image of immunophenotyping in the healthy group with gating strategies for monocyte subtypes (A), CD14⁺MHC II⁻, CD14⁺MHC II⁺, CD14⁻MHC II⁺; T lymphocyte subtypes (B), T helper lymphocytes (CD3⁺CD4⁺), activated T helper lymphocytes (CD3⁺CD4⁺CD25⁺), cytotoxic T lymphocytes (CD3⁺CD8⁺), activated cytotoxic T lymphocytes (CD3⁺CD8⁺CD25⁺), double negative T cells (CD4⁻CD8⁻), double positive T cells (CD4⁺CD8⁺); regulatory T lymphoyctes (C), CD4⁺FoxP3⁺CD25⁺; Natural killer cells (D); CD21⁻CD5⁻ CD3⁻, CD21⁻CD5^dim^ CD3⁻, CD21⁻CD5^dim^CD3⁺

Abbreviations: CD, cluster of differentiation; FSC, forward scatter; SSC, side scatter

**Supplementary figure 2:** Representative image of immunophenotyping in the preclinical group with gating strategies for monocyte subtypes (A), CD14⁺MHC II⁻, CD14⁺MHC II⁺, CD14⁻MHC II⁺; T lymphocyte subtypes (B), T helper lymphocytes (CD3⁺CD4⁺), activated T helper lymphocytes (CD3⁺CD4⁺CD25⁺), cytotoxic T lymphocytes (CD3⁺CD8⁺), activated cytotoxic T lymphocytes (CD3⁺CD8⁺CD25⁺), double negative T cells (CD4⁻CD8⁻), double positive T cells (CD4⁺CD8⁺); regulatory T lymphoyctes (C), CD4⁺FoxP3⁺CD25⁺; Natural killer cells (D); CD21⁻CD5⁻ CD3⁻, CD21⁻CD5^dim^ CD3⁻, CD21⁻CD5^dim^CD3⁺

Abbreviations: CD, cluster of differentiation; FSC, forward scatter; SSC, side scatter

**Supplementary figure 3:** Representative image of immunophenotyping in the compensated CHF group with gating strategies for monocyte subtypes (A), CD14⁺MHC II⁻, CD14⁺MHC II⁺, CD14⁻MHC II⁺; T lymphocyte subtypes (B), T helper lymphocytes (CD3⁺CD4⁺), activated T helper lymphocytes (CD3⁺CD4⁺CD25⁺), cytotoxic T lymphocytes (CD3⁺CD8⁺), activated cytotoxic T lymphocytes (CD3⁺CD8⁺CD25⁺), double negative T cells (CD4⁻CD8⁻), double positive T cells (CD4⁺CD8⁺); regulatory T lymphoyctes (C), CD4⁺FoxP3⁺CD25⁺; Natural killer cells (D); CD21⁻CD5⁻ CD3⁻, CD21⁻CD5^dim^ CD3⁻, CD21⁻CD5^dim^CD3⁺

Abbreviations: CD, cluster of differentiation; CHF, congestive heart failure; FSC, forward scatter; SSC, side scatter

**Supplementary figure 4:** Representative image of immunophenotyping in the decompensated CHF group with gating strategies for monocyte subtypes (A), CD14⁺MHC II⁻, CD14⁺MHC II⁺, CD14⁻MHC II⁺; T lymphocyte subtypes (B), T helper lymphocytes (CD3⁺CD4⁺), activated T helper lymphocytes (CD3⁺CD4⁺CD25⁺), cytotoxic T lymphocytes (CD3⁺CD8⁺), activated cytotoxic T lymphocytes (CD3⁺CD8⁺CD25⁺), double negative T cells (CD4⁻CD8⁻), double positive T cells (CD4⁺CD8⁺); regulatory T lymphoyctes (C), CD4⁺FoxP3⁺CD25⁺; Natural killer cells (D); CD21⁻CD5⁻ CD3⁻, CD21⁻CD5^dim^ CD3⁻, CD21⁻CD5^dim^CD3⁺

Abbreviations: CD, cluster of differentiation; CHF, congestive heart failure; FSC, forward scatter; SSC, side scatter

**Supplementary figure 5:** Correlations of immune cells with cytokines and chemokines

Abbreviations: CD3⁺, T lymphocytes; CD3⁺CD25⁺, activated T lymphocytes; CD3⁺CD4⁺, T helper lymphocytes; CD3⁺CD4⁺CD25⁺, activated T helper lymphocytes; CD3⁺CD8⁺, cytotoxic T lymphocytes; CD3⁺CD8⁺CD25⁺, activated cytotoxic T lymphocytes; CD4⁺FoxP3⁺CD25⁺, regulatory T lymphocytes; CD3⁻CD21^+^, B lymphocytes; DNT, double negative T lymphocytes; DPT, double positive T lymphocytes; IL, interleukin; KC-like, keratinocyte chemotactic-like; MCP-1, monocyte chemoattractant protein 1; NK, natural killer cell; TNF-α, tumor necrosis factor α

**Supplementary figure 6:** Correlations of the blood markers CRP and NT-proBNP with immune cells, echocardiographic parameters, cytokines and chemokines

Abbreviations: A, late mitral inflow wave; CD3⁺, T lymphocytes; CD3⁺CD25⁺, activated T lymphocytes; CD3⁺CD4⁺, T helper lymphocytes; CD3⁺CD4⁺CD25⁺, activated T helper lymphocytes; CD3⁺CD8⁺, cytotoxic T lymphocytes; CD3⁺CD8⁺CD25⁺, activated cytotoxic T lymphocytes; CD4⁺FoxP3⁺CD25⁺, regulatory T lymphocytes; CD3⁻CD21^+^, B lymphocytes; CRP, C-reactive protein; DNT, double negative T lymphocytes; DPT, double positive T lymphocytes; E, early mitral inflow wave; E/A, early to late mitral flow ratio; IL, interleukin; KC-like, keratinocyte chemotactic-like; LA/Ao, left atrial to aortic ratio; LAN, left atrial diameter in long axis right parasternal view normalized for body weight (cm/kg⁰∙³⁰⁹); LVIDdN, left ventricular end‐diastolic diameter in M-mode, normalized for body weight (cm/kg⁰∙²⁹⁹); LVIDsN, left ventricular end‐systolic diameter in M-mode, normalized for body weight (cm/kg⁰∙³⁸⁷); MCP-1, monocyte chemoattractant protein 1; NK, natural killer cell; NT-proBNP, N-terminal pro-B-type natriuretic peptide; TNF-α, tumor necrosis factor α

**Supplementary figure 7:** Correlations of immune cells with echocardiographic parameters

Abbreviations: A, late mitral inflow wave; CD3⁺, T lymphocytes; CD3⁺CD25⁺, activated T lymphocytes; CD3⁺CD4⁺, T helper lymphocytes; CD3⁺CD4⁺CD25⁺, activated T helper lymphocytes; CD3⁺CD8⁺, cytotoxic T lymphocytes; CD3⁺CD8⁺CD25⁺, activated cytotoxic T lymphocytes; CD4⁺FoxP3⁺CD25⁺, regulatory T lymphocytes; CD3⁻CD21^+^, B lymphocytes; CRP, C-reactive protein; DNT, double negative T lymphocytes; DPT, double positive T lymphocytes; E, early mitral inflow wave; E/A, early to late mitral flow ratio; LA/Ao, left atrial to aortic ratio; LAN, left atrial diameter in long axis right parasternal view normalized for body weight (cm/kg⁰∙³⁰⁹); LVIDdN, left ventricular end‐diastolic diameter in M-mode, normalized for body weight (cm/kg⁰∙²⁹⁹); LVIDsN, left ventricular end‐systolic diameter in M-mode, normalized for body weight (cm/kg⁰∙³⁸⁷); NK, natural killer cell
