# Supplementary material for: Immune response in dogs with myxomatous mitral valve disease: insights into monocyte and lymphocyte subtypes and natural killer cells
Source: J Vet Intern Med. 2026 Feb 25;40(1):aalag028. doi: 10.1093/jvimsj/aalag028 (PMC12935012; doi:10.1093/jvimsj/aalag028)
Supplement: aalag028_Supplemental_Files [file aalag028_supplemental_files.zip › Supplementary_material_1_clean_aalag028.docx]

**General Linear and Censored Regression Models**

Where between-group differences were present, the effect of confounding variables was examined. To control for confounding (sex and age), a general linear or censored regression model was fitted. Given the asymmetry of many variables, the data were log-transformed and the estimates presented accordingly. Heteroscedasticity was tested and a weighted general linear model was fitted as a sensitivity analysis. As the weighted general linear model produced results consistent with the simpler models, we report the results of the latter. However, for CRP (mg/L), a Gamma GLM with a log link was applied to better account for severe asymmetry. As a sensitivity analysis, we also fitted a weighted Gamma GLM in which observations with higher fitted means were downweighted. Results were consistent with the primary Gamma GLM, and therefore only the unweighted results are reported. For models where the outcome was cytokine concentration, a censored regression¹ was applied to account for samples with values below the detection limit, which were treated as left-censored data. The same global model was constructed using various parameters as the dependent variable, with group (healthy, preclinical, stable, and unstable CHF), sex (confounder), and age (confounder) as predictors. Later post-hoc pairwise comparisons between groups were conducted using estimated marginal means,² applying Tukey’s correction for multiple comparisons and are reported in text. For interpretability, results from transformed models are presented in the main text as back-transformed ratios of geometric means (GMRs).

**Table 1**

*GLM results for log(NT-proBNP (pmol/L) with Group, Age, and Sex as Predictors*

| *Explanatory Variable* | *Estimate (SE)* | *t-value* | *p-value* | *95% CI* |
| --- | --- | --- | --- | --- |
| (Intercept) | 6.2917 (1.5543) | 4.048 | <0.001 | 3.245 – 9.338 |
| Group Preclinical | 0.2003 (0.2008) | 0.997 | 0.319 | -0.193 – 0.594 |
| Group Stable CHF | 1.0805 (0.2226) | 4.855 | <0.001 | 0.644 – 1.517 |
| Group Unstable CHF | 1.5660 (0.2143) | 7.306 | <0.001 | 1.146 – 1.986 |
| Age | 0.0070 (0.0021) | 3.360 | 0.001 | 0.003 – 0.011 |
| Sex | -0.0263 (0.0655) | -0.402 | 0.689 | -0.155 – 0.102 |

**Table 2**

*Gamma GLM (log link) results for CRP (mg/L) with Group, Age, and Sex as Predictors*

| *Explanatory Variable* | *Estimate (SE)* | *z-value* | *p-value* | *95% CI* |
| --- | --- | --- | --- | --- |
| (Intercept) | 0.2392 (3.1411) | -0.455 | 0.649 | 0.001 – 112.855 |
| Group Preclinical | 0.9323 (0.3148) | -0.223 | 0.824 | 0.503 – 1.728 |
| Group Stable CHF | 3.9910 (0.4774) | 2.899 | **0.004** | 1.566 – 10.173 |
| Group Unstable CHF | 7.8746 (0.6196) | 3.331 | **0.001** | 2.338 – 26.524 |
| Age | 1.0020 (0.0040) | 0.506 | 0.613 | 0.994 – 1.011 |
| Sex | 1.0846 (0.1321) | 0.615 | 0.539 | 0.837 – 1.405 |

**Table 3**

*GLM results for log(WBC (x10^9^/L)) with Group, Age, and Sex as Predictors*

| *Explanatory Variable* | *Estimate (SE)* | *t-value* | *p-value* | *95% CI* |
| --- | --- | --- | --- | --- |
| (Intercept) | 3.4760 (0.8990) | 3.867 | <0.001 | 1.714 – 5.238 |
| Group Preclinical | -0.0389 (0.1162) | -0.335 | 0.739 | -0.267 – 0.189 |
| Group Stable CHF | -0.0293 (0.1287) | -0.228 | 0.821 | -0.282 – 0.223 |
| Group Unstable CHF | 0.5198 (0.1240) | 4.193 | <0.001 | 0.277 – 0.763 |
| Age | 0.0006 (0.0012) | 0.473 | 0.638 | -0.002 – 0.003 |
| Sex | -0.0605 (0.0379) | -1.597 | 0.114 | -0.135 – 0.014 |

**Table 4**

*GLM results for log(Neutrophils (×10⁹/L)) with Group, Age, and Sex as Predictors*

| *Explanatory Variable* | *Estimate (SE)* | *t-value* | *p-value* | *95% CI* |
| --- | --- | --- | --- | --- |
| (Intercept) | 3.4122 (1.1048) | 3.088 | 0.003 | 1.247 – 5.578 |
| Group Preclinical | -0.1341 (0.1428) | -0.939 | 0.351 | -0.414 – 0.146 |
| Group Stable CHF | -0.1473 (0.1582) | -0.931 | 0.355 | -0.457 – 0.163 |
| Group Unstable CHF | 0.5378 (0.1524) | 3.530 | 0.001 | 0.239 – 0.836 |
| Age | 0.0017 (0.0015) | 1.153 | 0.253 | -0.001 – 0.005 |
| Sex | -0.0793 (0.0465) | -1.703 | 0.093 | -0.170 – 0.012 |

**Table 5**

*GLM results for log(Monocytes (%)) with Group, Age, and Sex as Predictors*

| *Explanatory Variable* | *Estimate (SE)* | *t-value* | *p-value* | *95% CI* |
| --- | --- | --- | --- | --- |
| (Intercept) | 0.4764 (0.8428) | 0.565 | 0.574 | -1.176 – 2.128 |
| Group Preclinical | -0.3405 (0.1089) | -3.127 | 0.003 | -0.554 – -0.127 |
| Group Stable CHF | 0.0218 (0.1207) | 0.180 | 0.857 | -0.215 – 0.258 |
| Group Unstable CHF | 0.0017 (0.1162) | 0.015 | 0.988 | -0.226 – 0.230 |
| Age | 0.0046 (0.0011) | 4.029 | <0.001 | 0.002 – 0.007 |
| Sex | 0.0254 (0.0355) | 0.714 | 0.477 | -0.044 – 0.095 |

**Table 6**

*GLM results for log(Monocytes (×10⁹/L)) with Group, Age, and Sex as Predictors*

| *Explanatory Variable* | *Estimate (SE)* | *t-value* | *p-value* | *95% CI* |
| --- | --- | --- | --- | --- |
| (Intercept) | -0.6896 (1.2632) | -0.546 | 0.585 | -3.165 – 1.786 |
| Group Preclinical | -0.3710 (0.1632) | -2.273 | 0.023 | -0.691 – -0.051 |
| Group Stable CHF | -0.0072 (0.1809) | -0.040 | 0.968 | -0.362 – 0.347 |
| Group Unstable CHF | 0.5233 (0.1742) | 3.004 | 0.003 | 0.182 – 0.865 |
| Age | 0.0052 (0.0017) | 3.043 | 0.002 | 0.002 – 0.009 |
| Sex | -0.0338 (0.0532) | -0.635 | 0.525 | -0.138 – 0.070 |

**Table 7**

*GLM results for Lymphocytes (%) with Group, Age, and Sex as Predictors*

| *Explanatory Variable* | *Estimate (SE)* | *t-value* | *p-value* | *95% CI* |
| --- | --- | --- | --- | --- |
| (Intercept) | 5.2908 (20.2798) | 0.261 | 0.795 | -34.457 – 45.038 |
| Group Preclinical | 6.5573 (2.6203) | 2.502 | 0.012 | 1.422 – 11.693 |
| Group Stable CHF | 5.3438 (2.9038) | 1.840 | 0.066 | -0.348 – 11.035 |
| Group Unstable CHF | -1.2600 (2.7966) | -0.451 | 0.652 | -6.741 – 4.221 |
| Age | -0.0869 (0.0273) | -3.184 | 0.001 | -0.140 – -0.033 |
| Sex | 1.1253 (0.8543) | 1.317 | 0.188 | -0.549 – 2.800 |

**Table 8**

*GLM results for log(NLR) with Group, Age, and Sex as Predictors*

| *Explanatory Variable* | *Estimate (SE)* | *t-value* | *p-value* | *95% CI* |
| --- | --- | --- | --- | --- |
| (Intercept) | 2.7075 (1.4320) | 1.891 | 0.059 | -0.099 – 5.514 |
| Group Preclinical | -0.5033 (0.1850) | -2.720 | 0.007 | -0.866 – -0.141 |
| Group Stable CHF | -0.5128 (0.2050) | -2.501 | 0.012 | -0.915 – -0.111 |
| Group Unstable CHF | 0.0548 (0.1975) | 0.277 | 0.782 | -0.332 – 0.442 |
| Age | 0.0062 (0.0019) | 3.209 | 0.001 | 0.002 – 0.010 |
| Sex | -0.0903 (0.0603) | -1.497 | 0.134 | -0.209 – 0.028 |

**Table 9**

*GLM results for log(CD3⁺CD25⁺ (%)) with Group, Age, and Sex as Predictors*

| *Explanatory Variable* | *Estimate (SE)* | *t-value* | *p-value* | *95% CI* |
| --- | --- | --- | --- | --- |
| (Intercept) | 2.4441 (1.0054) | 2.431 | 0.015 | 0.473 – 4.415 |
| Group Preclinical | 0.1820 (0.1299) | 1.401 | 0.161 | -0.073 – 0.437 |
| Group Stable CHF | -0.2955 (0.1440) | -2.053 | 0.040 | -0.578 – -0.013 |
| Group Unstable CHF | 0.0702 (0.1386) | 0.506 | 0.613 | -0.202 – 0.342 |
| Age | 0.0027 (0.0014) | 1.990 | 0.047 | 0.000 – 0.005 |
| Sex | 0.0137 (0.0424) | 0.323 | 0.747 | -0.069 – 0.097 |

**Table 10**

*GLM results for log(CD3⁺CD4⁺CD25⁺ (%))with Group, Age, and Sex as Predictors*

| *Explanatory Variable* | *Estimate (SE)* | *t-value* | *p-value* | *95% CI* |
| --- | --- | --- | --- | --- |
| (Intercept) | 3.3689 (0.9379) | 3.592 | <0.001 | 1.531 – 5.207 |
| Group Preclinical | 0.0316 (0.1212) | 0.261 | 0.795 | -0.206 – 0.269 |
| Group Stable CHF | -0.8866 (0.1343) | -6.602 | <0.001 | -1.150 – -0.623 |
| Group Unstable CHF | -0.0886 (0.1293) | -0.685 | 0.494 | -0.342 – 0.165 |
| Age | 0.0028 (0.0013) | 2.195 | 0.029 | 0.000 – 0.005 |
| Sex | -0.0231 (0.0395) | -0.584 | 0.560 | -0.101 – 0.054 |

**Table 11**

*GLM results for log(DNT (%))with Group, Age, and Sex as Predictors*

| *Explanatory Variable* | *Estimate (SE)* | *t-value* | *p-value* | *95% CI* |
| --- | --- | --- | --- | --- |
| (Intercept) | 1.9380 (1.0350) | 1.872 | 0.062 | -0.091 – 3.967 |
| Group Preclinical | 0.3745 (0.1337) | 2.800 | 0.006 | 0.112 – 0.637 |
| Group Stable CHF | 0.1291 (0.1482) | 0.871 | 0.385 | -0.161 – 0.420 |
| Group Unstable CHF | 0.3753 (0.1427) | 2.629 | 0.010 | 0.096 – 0.655 |
| Age | -0.0011 (0.0014) | -0.806 | 0.421 | -0.004 – 0.002 |
| Sex | 0.0292 (0.0436) | 0.670 | 0.504 | -0.056 – 0.115 |

**Table 12**

*GLM results for log(Monocytes (%))with Group, Age, and Sex as Predictors*

| *Explanatory Variable* | *Estimate (SE)* | *t-value* | *p-value* | *95% CI* |
| --- | --- | --- | --- | --- |
| (Intercept) | 1.1707 (1.0862) | 1.078 | 0.281 | -0.958 – 3.300 |
| Group Preclinical | -0.6853 (0.1404) | -4.883 | <0.001 | -0.960 – -0.410 |
| Group Stable CHF | -0.2268 (0.1555) | -1.458 | 0.145 | -0.532 – 0.078 |
| Group Unstable CHF | -0.2185 (0.1498) | -1.458 | 0.145 | -0.512 – 0.075 |
| Age | 0.0074 (0.0015) | 5.084 | <0.001 | 0.005 – 0.010 |
| Sex | 0.0038 (0.0458) | 0.083 | 0.934 | -0.086 – 0.093 |

**Table 13**

*Censored regression model results for KC-like (pg/mL)with Group, Age, and Sex as Predictors*

| *Explanatory Variable* | *Estimate (SE)* | *z-value* | | *p-value* | *95% CI* |
| --- | --- | --- | --- | --- | --- |
| (Intercept) | 253.0208 (343.6182) | | 0.736 | 0.464 | -420.458 – 926.500 |
| Group [Preclinical] | -6.6171 (44.4661) | | -0.149 | 0.882 | -93.769 – 80.535 |
| Group [Stable CHF] | 20.8893 (49.2467) | | 0.424 | 0.673 | -75.632 – 117.411 |
| Group [Unstable CHF] | 108.0007 (47.4418) | | **2.276** | **0.026** | 15.017 – 200.985 |
| Age | 0.6372 (0.4627) | | 1.377 | 0.173 | -0.269 – 1.544 |
| Sex | -7.6656 (14.4793) | | -0.529 | 0.598 | -36.044 – 20.713 |
| logSigma | 4.7914 (0.0792) | | **60.481** | **<0.001** | 4.636 – 4.946 |

**Table 14**

*Censored regression model results for MCP-1 (pg/mL)with Group, Age, and Sex as Predictors*

| *Explanatory Variable* | *Estimate (SE)* | *z-value* | | *p-value* | *95% CI* |
| --- | --- | --- | --- | --- | --- |
| (Intercept) | -1291.5061 (1603.1413) | | -0.806 | 0.423 | -4433.602 – 1850.588 |
| Group [Preclinical] | -69.4022 (207.5031) | | -0.334 | 0.739 | -476.100 – 337.296 |
| Group [Stable CHF] | -13.3403 (229.7643) | | -0.058 | 0.954 | -463.670 – 436.990 |
| Group [Unstable CHF] | 778.6099 (221.3664) | | **3.517** | **0.001** | 344.740 – 1212.480 |
| Age | 3.8899 (2.1602) | | 1.801 | 0.076 | -0.344 – 8.123 |
| Sex | 57.0314 (67.5611) | | 0.844 | 0.401 | -75.386 – 189.448 |
| logSigma | 6.3315 (0.0791) | | **80.013** | **<0.001** | 6.174 – 6.487 |

**Abbreviations:** CD3⁺CD25⁺, activated T lymphocytes; CD3⁺CD4⁺CD25⁺, activated T helper lymphocytes; CHF, congestive heart failure; CI, confidence interval; CRP, C-reactive protein; DF, degrees of freedom DNT, double negative T lymphocytes; IL, interleukin; KC-like, keratinocyte chemotactic-like; MCP-1, monocyte chemoattractant protein 1; NLR, neutrophil to lymphocyte ratio; NT-proBNP, N-terminal pro-B-type natriuretic peptide; SE, standard error; WBC, total white blood cell count

**Reference:**

1. Henningsen A (2024). censReg: Censored Regression (Tobit) Models. R package version 0.5-39, <https://r-forge.r-project.org/projects/sampleselection>.
2. Lenth R (2025). emmeans: Estimated Marginal Means, aka Least-Squares Means. R package version 1.10.7, <https://rvlenth.github.io/emmeans/>.
