# Supplementary figures and images for: Immune response in dogs with myxomatous mitral valve disease: insights into monocyte and lymphocyte subtypes and natural killer cells
Source: J Vet Intern Med. 2026 Feb 25;40(1):aalag028. doi: 10.1093/jvimsj/aalag028 (PMC12935012; doi:10.1093/jvimsj/aalag028)

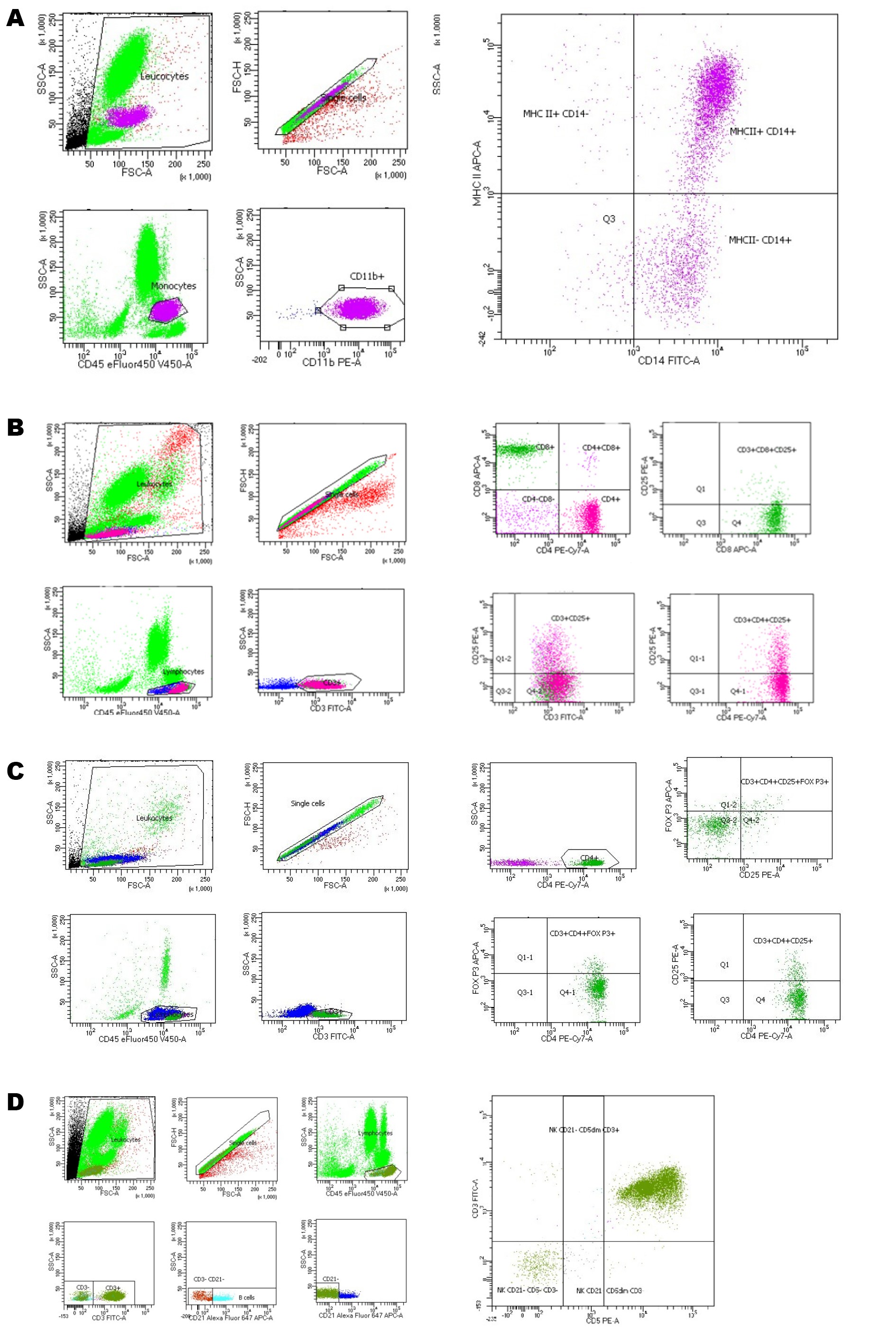

Supplement: aalag028_Supplemental_Files [file aalag028_supplemental_files.zip › Supp_figure_1_-_healthy_group_aalag028.jpg]

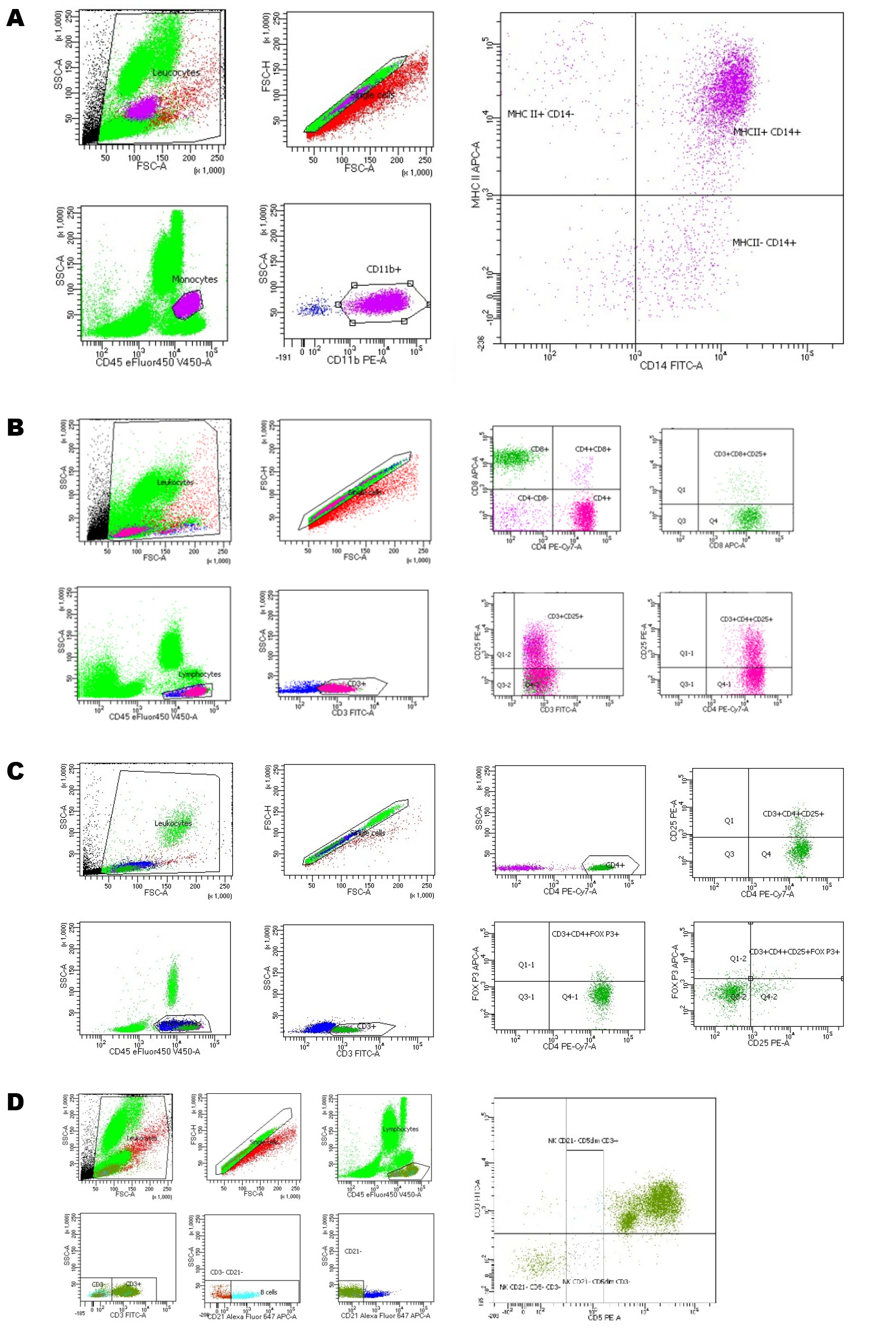

Supplement: aalag028_Supplemental_Files [file aalag028_supplemental_files.zip › Supp_figure_2_-_preclinical_group_aalag028.jpg]

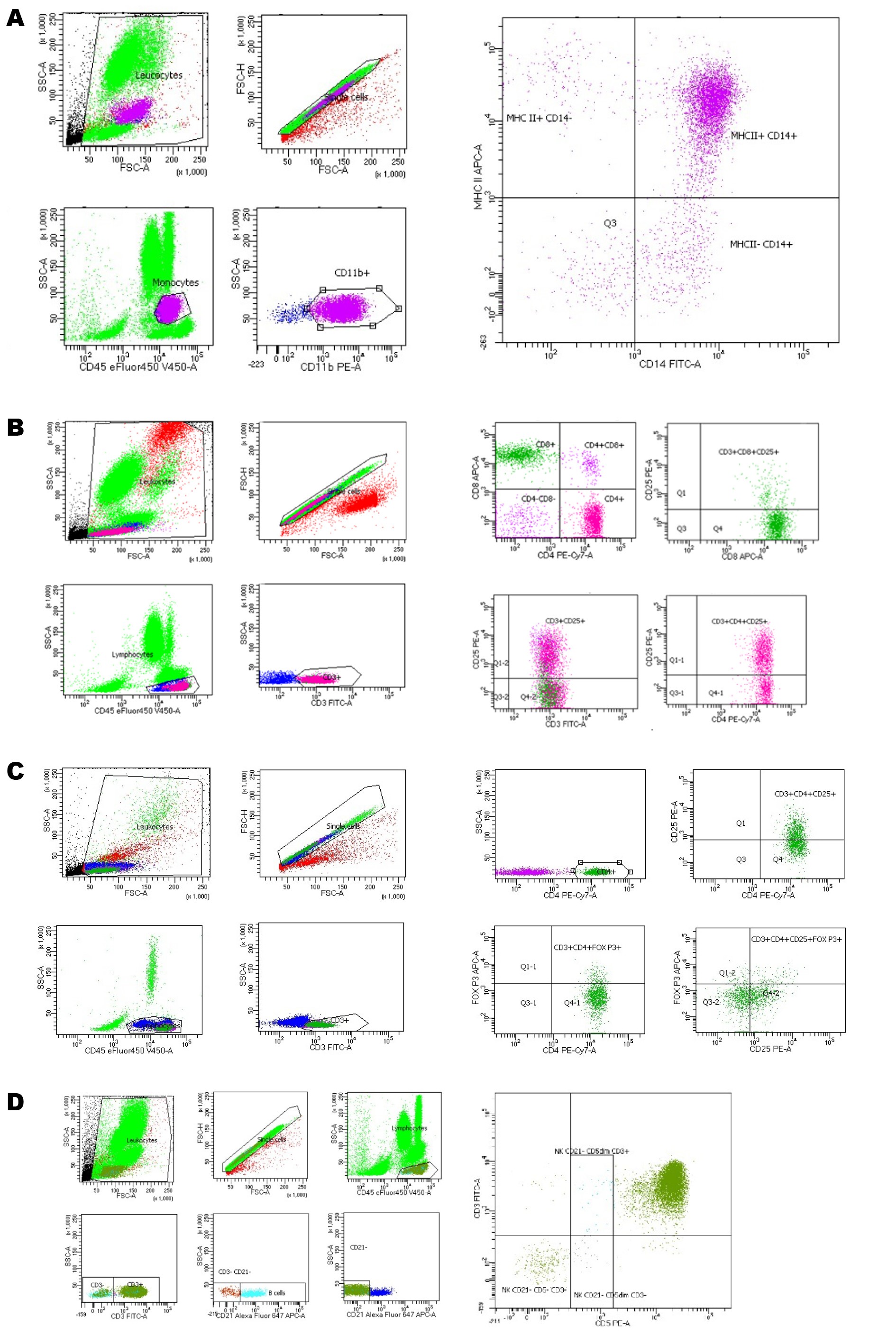

Supplement: aalag028_Supplemental_Files [file aalag028_supplemental_files.zip › Supp_figure_3_-_stable_CHF_group_aalag028.jpg]

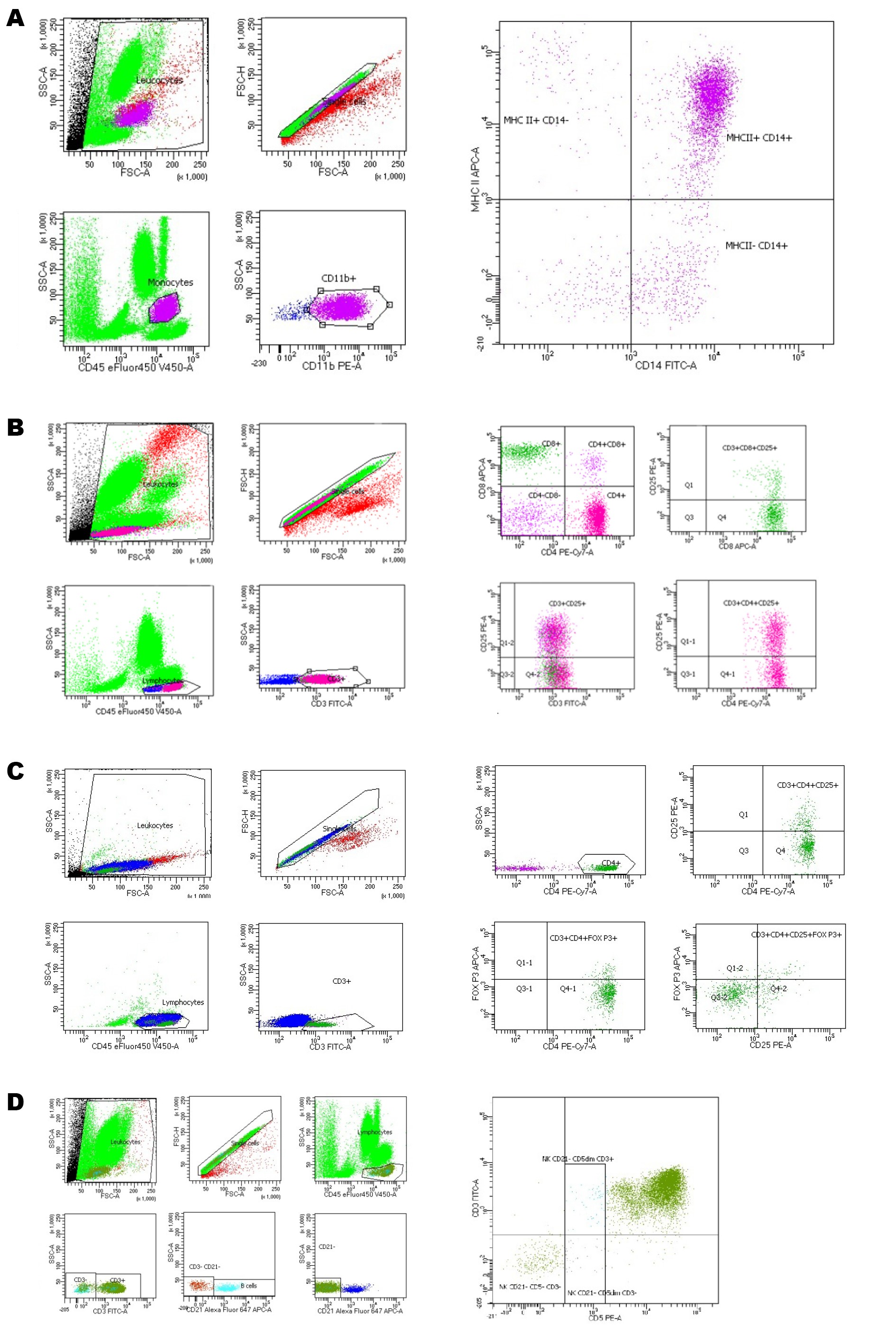

Supplement: aalag028_Supplemental_Files [file aalag028_supplemental_files.zip › Supp_figure_4_-_unstable_CHF_group_aalag028.jpg]

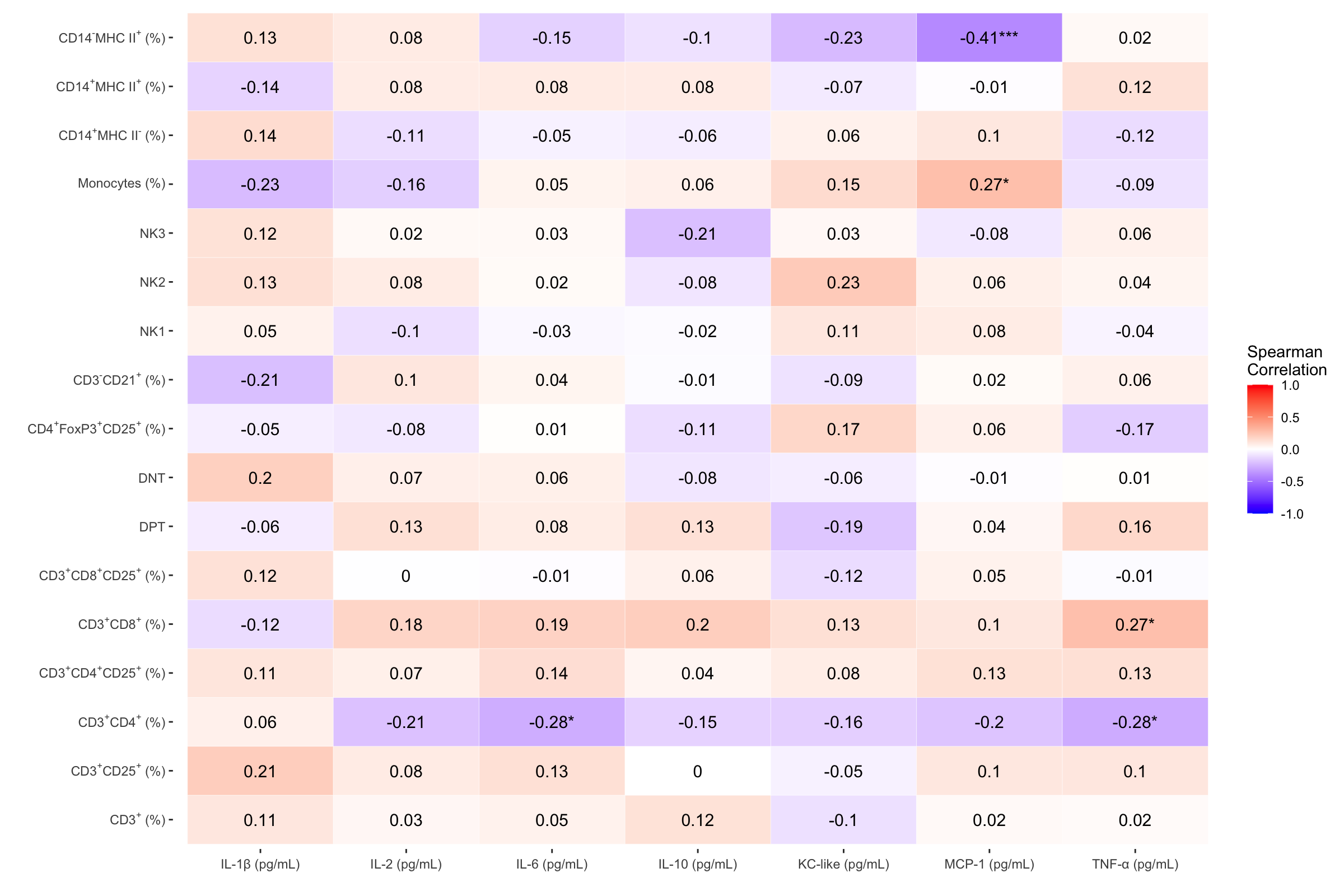

Supplement: aalag028_Supplemental_Files [file aalag028_supplemental_files.zip › Supp_figure_5_aalag028.tiff]

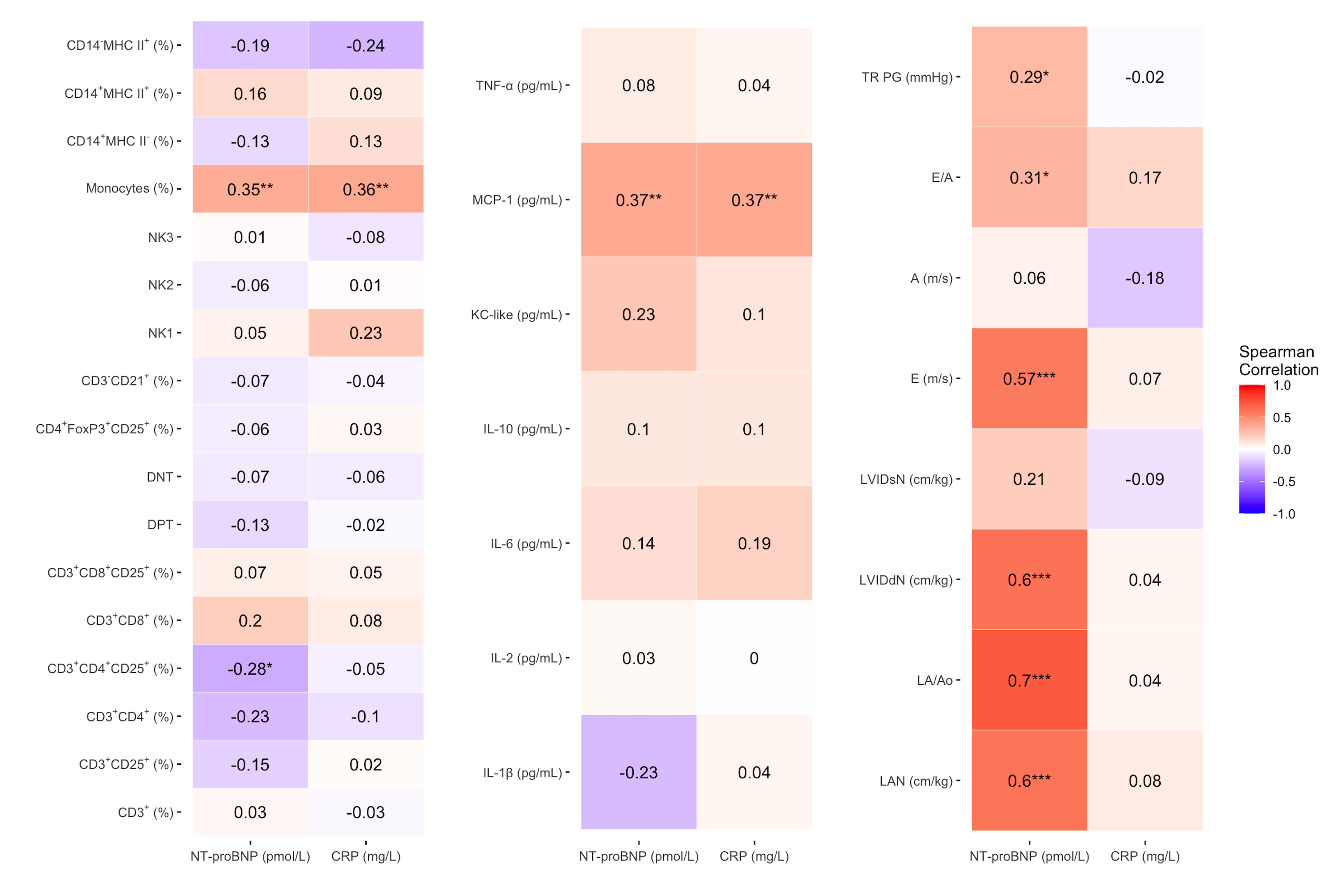

Supplement: aalag028_Supplemental_Files [file aalag028_supplemental_files.zip › Supp_figure_6_aalag028.tiff]

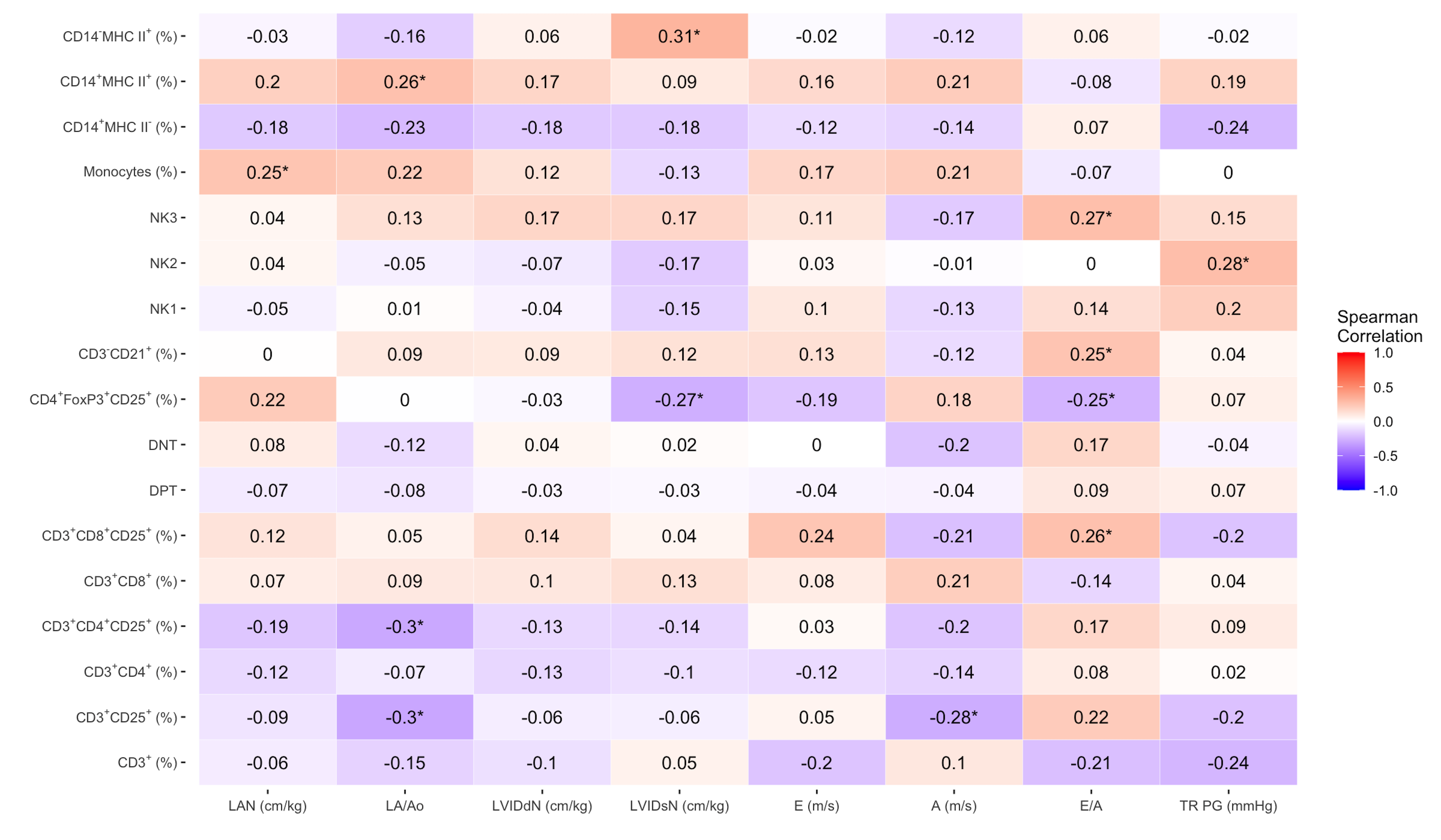

Supplement: aalag028_Supplemental_Files [file aalag028_supplemental_files.zip › Supp_figure_7_aalag028.tiff]
